# Hilltopping influences spatial dynamics in a patchy population of tiger moths

**DOI:** 10.1098/rspb.2022.0505

**Published:** 2022-06-08

**Authors:** Adam Pepi, Patrick Grof-Tisza, Marcel Holyoak, Richard Karban

**Affiliations:** ^1^ Department of Entomology and Nematology, University of California Davis, CA, USA; ^2^ Department of Environmental Science and Policy, University of California Davis, CA, USA; ^3^ Department of Biology, Tufts University, MA, USA; ^4^ Institute of Biology, University of Neuchâtel, Switzerland

**Keywords:** connectivity, metapopulation, hilltopping, dispersal, tiger moth, Bodega Marine Lab

## Abstract

Dispersal is a key driver of spatial population dynamics. Dispersal behaviour may be shaped by many factors, such as mate-finding, the spatial distribution of resources, or wind and currents, yet most models of spatial dynamics assume random dispersal. We examined the spatial dynamics of a day-flying moth species (*Arctia virginalis*) that forms mating aggregations on hilltops (hilltopping) based on long-term adult and larval population censuses. Using time-series models, we compared spatial population dynamics resulting from empirically founded hilltop-based connectivity indices and modelled the interactive effects of temperature, precipitation and density dependence. Model comparisons supported hilltop-based connectivity metrics including hilltop elevation over random connectivity, suggesting an effect of hilltopping behaviour on dynamics. We also found strong interactive effects of temperature and precipitation on dynamics. Simulations based on fitted time-series models showed lower patch occupancy and regional synchrony, and higher colonization and extinction rates when hilltopping was included, with potential implications for the probability of persistence of the patch network. Overall, our results show the potential for dispersal behaviour to have important effects on spatial population dynamics and persistence, and we advocate the inclusion of such non-random dispersal in metapopulation models.

## 1. Introduction

Dispersal plays a critical role in the dynamics of spatially structured populations [1,2]. It can maintain genetic diversity, rescue declining populations and allow recolonization of those that are locally extinct [3–5]. For extinction-prone populations, network-wide extinction risk is spread through loosely linked patches by infrequent dispersal events [3,4]. Conversely, a patch-network tightly linked through dispersal is at greater risk of network-wide collapse due to synchrony of local population dynamics. Landscape ‘connectivity’ is the broadly defined metric (reviewed by Kindlmann & Burel [6]) used to quantify the strength of these linkages [7]. Because connectivity measurements are intuitive and linked to population persistence, they are regularly used in reserve design and conservation planning to mitigate the effects of global change [8].

Despite their widespread application, the inclusion of connectivity metrics in patch-based models has historically provided poor predictions of patch occupancy [9]. This is in part due to the simplifying assumptions of such models. For example, many classic connectivity metrics assume random movement, where successful immigration into neighbouring patches is based solely on interpatch distance and dispersal ability [10,11]. However, empirical investigations of dispersal have repeatedly demonstrated that the likelihood of an individual arriving at any particular patch is far from random [12]; resource availability, conspecific densities, and densities of natural enemies can affect emigration and immigration decisions [13,14]. Moreover, the transition through matrix habitat between suitable patches can be limiting and is often influenced by landscape features. For example, topography can impede movement of some species [15] or attract individuals of other species that lek on hilltops, thereby creating barriers or corridors for dispersing individuals [16,17]. Because of the challenges of empirically measuring dispersal behaviour though observation, telemetry or mark-recapture, it is rarely done and even more seldomly incorporated into patch-based models.

The overarching aim of this study was to assess the implications of non-random dispersal for spatially structured populations. To achieve this, we studied the spatial and temporal dynamics of a patchily structured, diurnal moth population (*Arctia virginalis*; Lepidoptera: Erebidae) that exhibits hilltopping behaviour. Hilltopping is a common mate-locating strategy, where flying insects aggregate on sites of topographical prominence like hilltops and ridges to increase the likelihood of encountering conspecifics of the opposite sex [18,19]. Because hilltopping behaviour constrains movement through hilltop mating aggregations, it is a useful means of studying the consequences of non-random dispersal [16,20–22]. In *A. virginalis*, caterpillars pupate near natal patches, eclose as adults and move to nearby hilltop locations, from which adults disperse to larval patches and oviposit [23]. Adults often disperse to and oviposit in larval patches different from their natal patches since frequent patch colonizations and extinctions occur in this species [24]. In our previous work, a landscape connectivity index was developed that incorporated hilltopping behaviour [24] based on a mark-recapture study by the same authors [25]. However, the usefulness (or validity) of this connectivity index relative to traditional metrics was not tested. In the present study, we specifically compared the effects of three different connectivity indices, two of which incorporated the hilltopping behaviour of *A. virginalis*, using 5 additional years of data.

We focused specifically on the population-level consequences of hilltopping, by comparing the dynamical effects of three different connectivity indices, two of which incorporated the hilltopping behaviour of *A. virginalis*. First, using survey data, we examined the relationships between adult moth and caterpillar abundances in larval patches and hilltops, which form the empirical foundation for behaviourally based connectivity indices. We also used time-series models of caterpillar data in survey patches to compare traditional and behaviourally based connectivity indices as predictors of population dynamics. Lastly, we constructed simulation models based on parametrized time-series models using three connectivity indices to compare the consequences of random versus non-random dispersal on patch dynamics.

## 2. Methods

### (a) Overview

We conducted our analyses using long-term annual census data of caterpillar counts, and more irregular data of adult counts on hilltops. First, we examined the empirical relationship between (i) spring caterpillar counts in patches and summer moth counts on hilltops in the same year and (ii) summer moth counts on hilltops and caterpillar counts in patches in the following spring. Next, using these results, we developed two novel landscape connectivity indices, one including hilltops, and another including hilltops and the elevation of hilltops. We then conducted time-series analyses of long-term data, allowing us to compare our alternative connectivity indices and test for effects non-random dispersal on spatial population dynamics. Finally, we conducted simulations based on our best-fit model to illustrate how dispersal through hilltops affected spatial dynamics in terms of patch occupancy, extinctions, colonizations and regional synchrony.

### (b) Caterpillar censuses and climate variables

**Figure 1.**
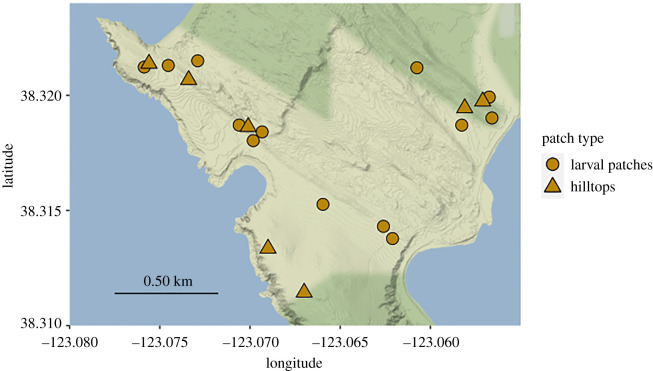
Map of Bodega Marine Reserve, showing location of surveyed larval patches (circles) and hilltops (triangles). (Online version in colour.)

We estimated *A. virginalis* caterpillar abundance at 12 habitat patches at BMR (figure 1) using 15 years of census data, from 2007 to 2021. Censuses were conducted in the last week of March each year, by counting the number of caterpillars observed on haphazardly selected *Lupinus arboreus* bushes of similar size at each patch (*n* = 10 bushes, 2007–2011; *n* = 15 bushes, 2011–2019; *n* = 20 bushes, 2020–2021; detailed methods in [26]). For analyses of the caterpillar censuses, we averaged over the number of bushes sampled and used the average density of caterpillars per bush at each site as our response variable. Climate variables were collected as part of monitoring efforts by Bodega Marine Reserve and the University of California Natural Reserve System. Precipitation was recorded with a rain gauge (Hydrological Services TB4 tipping bucket, Campbell Scientific, Ogden, UT), and temperature was recorded with a combined temperature and humidity sensor (CS215-L, Campbell Scientific, Ogden, UT). For analyses, total annual precipitation in the previous precipitation year (October 1–October 1) or average temperature in the previous dry season (June–October) were used. This was based on prior information that caterpillars are most vulnerable to adverse environmental conditions during the dry season months [27].

### (c) Moth censuses

Moths at seven known hilltop lek locations [23] were surveyed annually from 2010 to 2012, and from 2017 to 2019. Surveys were not practical from 2013 to 2016 and 2020 to 2021 due to low adult abundances. Surveys were conducted at variable dates each year during the early summer, from 27 May to 19 July. This range of dates was required because the phenology of pupation varied among years. Surveys were timed roughly a month after pupation was observed. Adult moths are relatively long lived, persisting up to three weeks in mark-recapture and caging studies [25].

### (d) Hilltops and larval patches

To investigate the determinants of hilltop aggregations, we analysed predictors of moth abundance on hilltops based on prior field studies, including connectivity to larval patches, and elevation of hilltops [23,25]. In previous work, we found that moths primarily were found on hilltops, and in greater abundance on higher elevation hilltops [23,25].

In these models, we used a patch connectivity index similar to that used by Hanski [28], which was of the form:

$$CI_{i,t}=\underset{i\neq j}{\sum }e^{-\alpha d_{ij}}A_{\;j,t},$$

where $CI_{i,t}$ is the connectivity index of patch *i* in year *t*, α is the inverse dispersal distance, which we set at 1 [24,25], $d_{ij}$ is the distance between patch *i* and patch *j,* and $A_{j,t}$ is log caterpillar or adult abundance in patch *j* in year *t*.

We constructed a set of Bayesian generalized linear mixed effect models of adult counts on hilltops, which we compared using WAIC, starting from the full model form:

$$\;\begin{array}{r} Y_{hilltop,j,t}∼Poisson(\alpha_{0}+\beta_{CI}\;*\;scale(ln(CI_{hilltop,j,t}))+\beta_{elevation}Elevation_{j}\\ +\beta_{CI\times E}\;*\;scale(ln(CI_{\;j,t}))Elevation_{j}+\alpha_{hilltop,j}+\alpha_{year,t}), \end{array}$$

in which $Y_{\;j,t}$ is the number of moths observed of hilltop *j* in year *t*, $CI_{hilltop,j,t}$ is a connectivity index of hilltop *j* in year *t* (logged and *z*-score scaled)*,* $Elevation_{j}$ is the elevation in ft of hilltop *j*, $\beta_{CI\times E}$ is an interaction between connectivity and elevation, $\alpha_{hilltop,j}$ is a random intercept for hilltop and $\alpha_{year,t}\;$is a random intercept for year. The connectivity indices were logged and scaled because the exponential nature of the indices created model fitting issues. Models were fit using brms [29], using vague normal priors for all terms (Normal(0,10)), random effects having a hyperprior of mean α and variance σ both using the default brms priors, Student's *t* (t(3,0,2.7)). Model structures without an interaction, with just elevation or connectivity, or an intercept-only model were compared with WAIC (table 1). This constituted all possible combinations of variables, with random effects retained in all models.

**Table 1.** Results of moth abundance model structure comparison, showing ΔWAIC and WAIC._._

model structure	ΔWAIC	WAIC
Connectivity+Elevation	0.00	290.58
Connectivity	11.01	301.58
Elevation	24.24	314.82
Intercept only	29.02	319.60
Connectivity+Elevation+Connectivity×Elevation	1.2 $\times 10^{60}$	1.2 $\times 10^{60}$

To examine the influence of adult dispersal from hilltops on larval populations, we analysed the relationship between moth abundance on hilltops and caterpillar abundance in larval patches in the subsequent year. We constructed a model of caterpillar counts in patches of the following form:

$$\begin{array}{rl} \ln(Y_{\;patch,i,t}+1) & ∼N(\alpha_{0}+\beta_{CI}\;*\;scale(ln(CI_{\;patch,i,t-1}))\\ & \;+\alpha_{\;patch,i}+\alpha_{year,t},\sigma ), \end{array}$$

in which $Y_{\;patch,i,t}$ is the average caterpillar count in patch *i* in year *t*, $CI_{\;patch,i,t-1}$ is a connectivity index of patch *i* relative to hilltops in year *t−1* (logged and *z*-score scaled)*,* $\alpha_{\;patch,i}$ is a random intercept for patch *i* and $\alpha_{year,t}\;$is a random intercept for year. Models were fit using brms, using vague normal priors for all terms (Normal(0,10)) except the variance and random effects, with random effects having a hyperprior of mean α and variance σ. Variance and hyperpriors were both the default brms priors, Student's t (t(3,0,2.5)). The full model was compared to an intercept-only model using WAIC.

### (e) Hilltopping connectivity indices

For our analyses, we developed two connectivity indices representing non-random dispersal behaviour of adult moths, one including hilltops, and a second also including elevation of hilltops. These connectivity indices represent a movement process in which moths move from natal patches to hilltops, mate, and then disperse to larval patches to oviposit. In the first index (HCI), only distance to hilltops is considered, whereas in the second (ECI) moths preferentially move to higher elevation hilltops. The construction of these indices was informed by *a priori* knowledge of adult movement behaviour [23,25] and empirical measurements of predictors of moth occurrence on hilltops (previous section).

Our hilltop-based connectivity index was of the form:

$$HCI_{i,t}=\underset{\;j=1}{\sum }e^{-\alpha d_{ij}}\;*\;scale\left(\underset{k=1}{\sum }e^{-\alpha d_{jk}}A_{k,t}\right),$$

where $HCI_{i,t}$ is the connectivity index of patch *i* in year *t*, α is the inverse mean dispersal distance, $d_{ij}$ is the distance between patch *i* and hilltop *j,* $d_{jk}$ is the distance between hilltop *j* and patch *k,* and $A_{k,t}$ is log caterpillar abundance in patch *k* in year *t.* This connectivity index measures connectivity of patches through hilltops and was thus hypothesized to be a better representation of the spatial dispersal pattern of *A. virginalis*.

We also developed a connectivity index representing non-random movement occurring through hilltops that incorporated hilltop elevation. This was parametrized using the best-fit model of moth abundance on hilltops (previous section) and was of the following form:

$$ECI_{i,t}=\underset{\;j=1}{\sum }e^{-\alpha d_{ij}}M_{\;j,t},$$

$$M_{\;j,t}=-0.89+1.27\;*\;scale(ln(CI_{j,t}))+0.02(Elevation_{j}),$$

$$CI_{\;j,t}=\underset{k=1}{\sum }e^{-\alpha d_{jk}}A_{k,t},$$

where $ECI_{i,t}$ is the connectivity index of patch *i* in year *t*, α is the inverse average dispersal distance, $d_{ij}$ is the distance between patch *i* and hilltop *j,* $M_{j,t}$ is log predicted moth count on hilltop *j* in year *t*, $CI_{j,t}$ is the connectivity index of hilltop *j* in year *t,* $Elevation_{j}$ is the elevation in metres of hilltop *j*, $d_{jk}$ is the distance between hilltop *j* and patch *k,* and $A_{k,t}$ is log caterpillar abundance in patch *k* in year *t.* This connectivity index measures connectivity of patches through hilltops, but with greater dispersal through higher hilltops, and was thus hypothesized to be a better representation of the spatial dispersal pattern of *A. virginalis* based on empirical findings from models of adult moth surveys (above sections). In this index, we used log predicted moths to allow for the use of the index in years and sites where counts were missing.

### (f) Caterpillar time-series models

To examine the effects of random versus non-random movement behaviour on caterpillar population dynamics, we constructed a series of time-series models including the alternative connectivity indices developed above. We modelled dynamics in each patch separately but linked patches through dispersal via connectivity terms. We used connectivity estimates rather than moth counts, because moth counts were only available for a few years. Previous work in this system found that precipitation had strong effects on dynamics [24], and that temperature affected dynamics of *A. virginalis* and its ant predator [30]. We therefore included precipitation and temperature in our models, along with the interactive effects of temperature and precipitation on population dynamics. We also included direct and delayed density dependence terms, intended to represent direct and delayed effects of baculovirus infection on dynamics that we have previously documented [31,32]. We constructed a series of models starting from the following full model structure:

$$\begin{array}{rl} X_{t,i} & ∼Normal(a_{0}+a_{1}X_{t-1,i}+a_{2}X_{t-2,i}+\beta_{\;precip}Precip_{t-1}\\ & \;+\beta_{temp}Temp_{t-1}+\beta_{\;precip\times temp}Precip_{t-1}Temp_{t-1}\\ & \;+\;\beta_{connectivity}Connectivity_{i,t-1}+\alpha_{\;patch,i},\;\sigma_{i}^{2}), \end{array}$$

where $X_{t,i}$ is the log caterpillar count in year *t* and patch *i*. The model includes an intercept representing population growth at low density $\left(a_{0}\right)$, direct density dependence $\left(a_{1}\right)$, delayed density dependence $\left(a_{2}\right)$, an effect of precipitation $\left(\beta_{\;precip}\right)$, an effect of temperature$\left(\beta_{temp}\right)$, an interaction effect between precipitation and temperature $\left(\beta_{\;precip\times temp}\right)$, an effect of connectivity $\left(\beta_{connectivity}\right)$ and a random intercept for patch ($\alpha_{\;patch,i}$). Variance was allowed to vary between patches ($\;\sigma_{i}^{2}$).

We compared models using WAIC that included all, or a subset, of parameters in the full model above (table 2). We included all autoregressive parameters and random effects in all models and generated all possible combinations of variables for the reduced models. Versions of all models using three different connectivity indices were compared: the standard patch connectivity index (CI), the hilltop-based connectivity matrix (HCI) or the hilltop-based connectivity index incorporating hilltop elevation (ECI; table 2). A prior of Normal(0,10) was used for all parameters except for the variance and random effects. For the variance, we used a Cauchy(0,5) prior, and for the random effect, we used a prior of $Normal(0,\sigma_{site})$ and a hyperprior for the variance $\sigma_{site}$ of Cauchy(0,5). Models were fit in Stan [33].

**Table 2.** Results of autoregressive model selection, showing ΔWAIC from best-fit model, WAIC values, ΔWAIC of the Hanksi connectivity index (CI) versus the elevation connectivity index (ECI) and ΔWAIC of the hilltopping connectivity index (HCI) versus the elevation connectivity index (ECI).

model structure	ΔWAIC	WAIC	CI−ECI	HCI−ECI
Precip+Temp+Precip×Temp+Connectivity	0.00	581.28	6.86	3.65
Precip+Temp+Precip×Temp	4.02	585.30	—	—
Precip+Temp+Connectivity	25.06	606.33	7.15	4.85
Precip+Temp	30.62	611.90	—	—
Temp+Connectivity	31.17	612.45	5.90	2.12
Temp	33.13	614.41	—	—
Precip+Connectivity	55.95	637.22	–5.73	2.59
Precip	59.07	640.35	—	—
Connectivity	73.89	655.17	–4.92	8.69

### (g) Simulation model

To assess the effects of non-random dispersal on spatial dynamics in this population, we conducted simulations using the best-fit time-series model (table 2). To conduct simulations, we used the observed starting values for each patch in 2007 and 2008 and drew random values for population sizes in each patch for the subsequent year from a Poisson distribution using a mean value calculated with the time-series model formula. Empirical values of temperature and precipitation were used, and connectivity indices were calculated based on population sizes at each patch in the current year and scaled using the mean and standard deviation of observed connectivity values. We conducted versions of the simulations using the standard patch connectivity index (CI), the hilltop connectivity index (HCI) and the elevation connectivity index (ECI). Ten thousand simulations were run over a 15-year period, and occupancy, extinction, colonization and regional synchrony (using package ncf [34]) were summarized and compared between the models using the different dispersal processes. Several individual simulations were visually inspected to make sure that simulated population densities were not outside of reasonable values.

## 3. Results

### (a) Hilltops and larval patches

**Figure 2.**
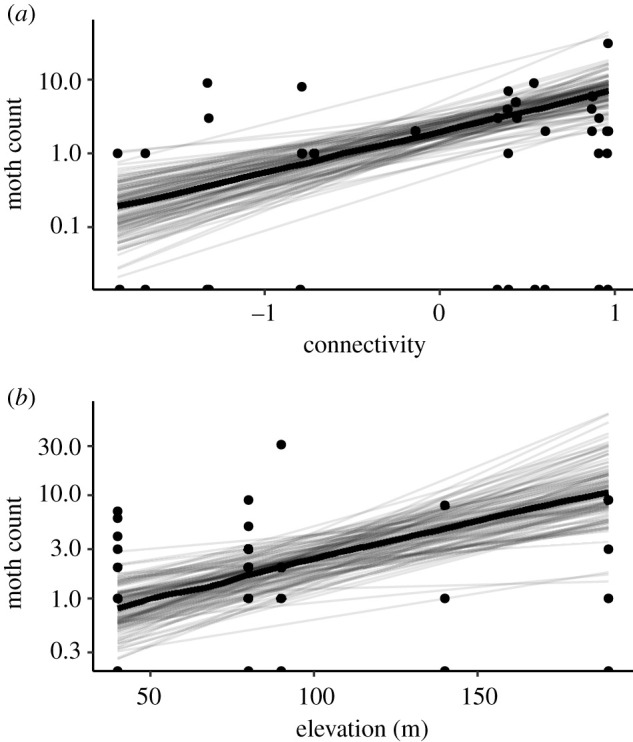
Best-fit model of moth abundance, showing relationship with (logged and scaled) connectivity to larval patches (*a*) and elevation in metres (*b*). Maximum *a posteriori* line is shown in wide black lines, plotted over 50 lines sampled from the posterior.

Connectivity between hilltops where mating occurred and larval food patches was a better predictor of adult abundance than caterpillar abundance. The best-fit model for adult moths on hilltops included connectivity to larval patches and elevation (table 1), both of which were good predictors of moth abundance (figure 2; $R_{m}^{2}=0.13$).

**Figure 3.**
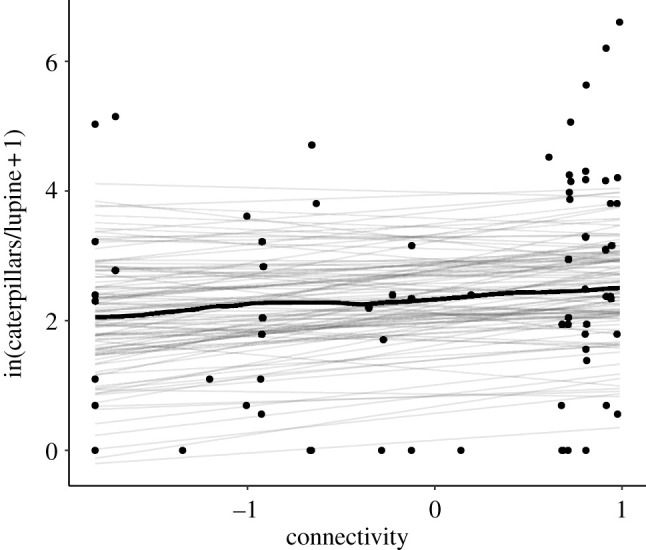
Model of caterpillar abundance, showing relationship with (logged and scaled) connectivity to moth hilltops. Maximum *a posteriori* line is shown in wide black lines, plotted over 50 lines sampled from the posterior.

Connectivity between moths on hilltops and patches of larval habitat was a poor predictor of caterpillar counts in patches in the subsequent year ($\beta_{connectivity}=0.18\;[95\text{\%};$ $HDPI:-0.22-0.59],\;R_{m}^{2}=0.014$, figure 3), and the model including connectivity had a similar fit to the intercept-only model (ΔWAIC=1.4).

### (b) Caterpillar time-series models

**Figure 4.**
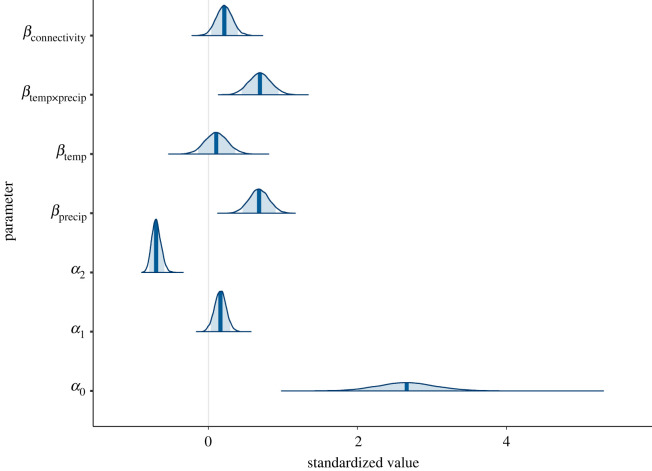
Posteriors densities from the best-fit autoregressive model (table 2), with 90% intervals shaded, density profiles extending to 99% intervals and maximum *a posteriori* estimates plotted with vertical lines. Beta values are covariates in the autoregressive model (precipitation, temperature, interaction term and connectivity), and alpha values are the model intercept, direct and delayed density dependence. (Online version in colour.)

Caterpillar dynamics were affected by abiotic conditions. The full model (table 2), including, precipitation, temperature, connectivity and an interaction between temperature and precipitation best fit the data (figure 4). For the best-fit model, the hilltop connectivity index that included hilltop elevation (ECI) was favoured over Hanski's connectivity index (CI) by ΔWAIC=6.86 and the hilltop connectivity index (HCI) by ΔWAIC=3.65 (table 2).

### (c) Simulation results

**Figure 5.**
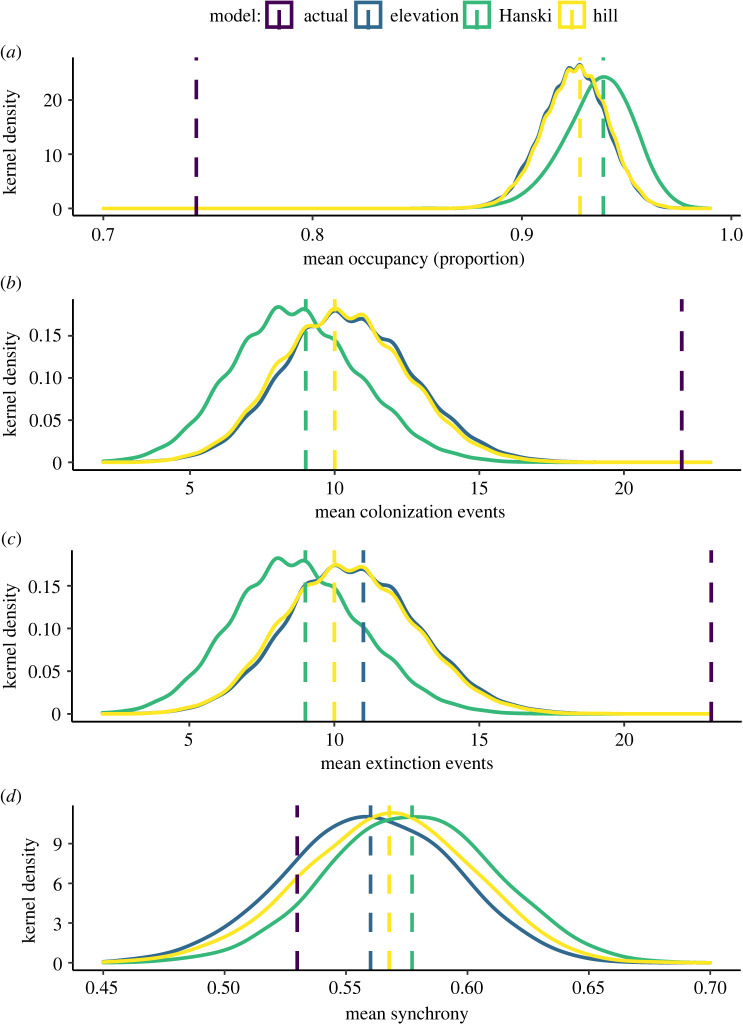
Connectivity simulation results, comparing occupancy (*a*), colonization (*b*), extinction (*c*) and regional synchrony (*d*) between the elevation, hilltop and Hanski models. (Online version in colour.)

In metapopulation simulations, the hilltop connectivity index (HCI) and the elevation connectivity index (ECI) had somewhat higher colonization and extinction rates and lower occupancy compared to the standard patch connectivity index (CI) (figure 5; electronic supplementary material, table S1), although simulated distributions overlapped substantially. The simulations with standard patch connectivity had higher regional synchrony than the hilltop or elevation connectivity models. For all metrics, hilltop-based connectivity simulation results had closer median values to observed values than other connectivity functions, although the actual observed values were quite different from the simulation results (figure 5).

## 4. Discussion

Overall, our results showed that hilltopping behaviour was a significant contributor to spatial dynamics in this population system. Empirically parametrized connectivity indices were significant predictors of population dynamics, and they were better predictors of dynamics than a standard connectivity index that did not include hilltopping (table 2). Simulations demonstrated that, compared to other forms of connectivity, hilltopping resulted in lower patch occupancy rates, higher extinction and colonization rates, and marginally lower regional synchrony (figure 5). Simulated outcomes from models including hilltopping were closer to observed results in the field than were results from simulations not including hilltopping, although all simulations results were relatively different from observed results. This is likely because our time-series model included fewer sources of variation than exist in the system. These could include generalist predation [35], which is likely to generate Allee effects and the increased extinction and colonization rates observed relative to simulations. Overall, our findings show that non-random dispersal behaviour can influence spatial population dynamics, as has been predicted previously based on behavioural studies [12,16].

We also found empirical support for our hilltopping connectivity indices from monitoring moth abundances on hilltops. Results from models of hilltops and larval patches were consistent with observations that moths fly to nearby hilltops to find mates, and once mated, fly to nearby larval habitat patches to lay eggs. This local bi-directional movement produced a positive relationship between larval patches and nearby hilltop moth counts (figure 2), although we did not find a clear relationship between hilltop moth counts and caterpillars in larval patches in the following year (figure 3). This lack of a relationship is likely because larval and adult density are imperfect predictors of the next stage, since there is mortality (e.g. due to disease [32] and predation [35]) during the intervening periods can be density dependent. Such density-dependent processes have high potential to remove any correlation between these stages, and therefore the effects of movement were likely substantially stronger before density-dependent mortality. In addition, the long period between adult surveys (May–July) and caterpillar surveys in the following year (March) includes the summer period when caterpillars are most affects by interannual climate variation [27]. Overall, our analyses of the relationship between caterpillars counts in patches and adult counts on hilltops provided an empirical basis for our hilltopping connectivity indices used in subsequent time-series analyses. Both empirical comparisons of abundances on hilltops and larval patches and time-series analyses found that connectivity indices that included hilltop elevation were better supported than those that did not (tables 1 and 2). This is in line with previous findings that moth abundance on hilltops was well predicted by elevation [25] and as predicted by simulations within the same landscape [22].

Time-series models suggested that the effects of connectivity were similar in magnitude or only slightly weaker than climate or density-dependent effects (figure 4). Simulations further illustrated differences in dynamics between random movement, and dispersal that occurred specifically through hilltops. Because movement through hilltops is non-random and spatially restricted, simulations that included dispersal through hilltops found lower occupancy and higher extinction and colonization rates. This suggests that turnover (extinction and colonization) of local populations distant from hilltops may be more frequent than would be predicted by random movement models. By contrast, simulations with hilltopping dispersal had lower regional synchrony which could instead increase the likelihood of regional persistence [36]. This lower regional synchrony is likely due to hilltops serving as local attractors, driving divergence of dynamics between clusters of patches around different hilltops.

This is the first empirical study demonstrating dynamical consequences of hilltopping in a patchy population, though previous work has shown effects of other modes of non-random dispersal on population dynamics [37]. Previous empirical and theoretical workers have hypothesized that non-random dispersal via hilltops will have strong effects on spatial population dynamics since these dynamics are heavily influenced by dispersal [16,21,22,38]. However, this question has not been tested directly: one study found that topographic prominence (i.e. hilltops) was a strong predictor of genetic differentiation in a hilltopping butterfly species (*Papilio machaon*), which would be an expected result of hilltops functioning as local attractors of population dynamics and dispersal [39].

We found strong interactive effects of temperature and precipitation on population dynamics, with higher population growth in wetter and warmer years (electronic supplementary material, figure S1). One potential explanation for this finding is that climate effects were mediated from the bottom up, via poor host-plant quality in years unsuitable for plant growth (hot and dry, cold and wet, etc.). Indeed, our current work suggests this may be the primary mechanism through which temperature and precipitation affect dynamics of early instar larvae in this population (Pepi, Grof-Tisza, Holyoak and Karban, unpublished data). This in agreement with other studies that inferred extinction and colonization dynamics were affected by climate-mediated changes in resource abundance or patch quality [24,40,41]. Considering that climate models predict that severe weather will become more frequent in California [42], finding that *A. virginalis* is sensitive to climatic variation has strong implications for the persistence of local populations; climate-mediated deterioration of habitat quality combined with fewer immigration events increases the likelihood of prolonged absences in isolated patches far from adult aggregations on hilltops. Though we demonstrated improved model performance when we used connectivity indices based on hilltoping behaviour, temperature and precipitation were found to be stronger drivers than connectivity of the spatial and population dynamics of *A. virginalis.* Despite the dominance of the area-and-isolation paradigm, this work illustrates the importance of considering climate effects when modelling the dynamics of patchy populations [24,40,43].

Most if not all organisms disperse in some predictable way whether deliberately like migrating birds or with less agency such as ballooning spiders adrift in the prevailing wind. Lekking mating systems, which have evolved in numerous taxonomic groups, are but one example of how a life-history trait may lead to non-random dispersal. Indeed, despite its widespread use, the assumption of random dispersal is rarely accurate. The advancement of landscape ecology has seen the development of better tools to predict movement patterns; however, many metapopulations models still rely on this outdated approach. Though our hilltopping connectivity index generated simulated results only modestly closer to observed results than a standard patch connectivity index, small differences may be important for species of conservation concern. Alarmingly, it is for these vulnerable species that metapopulations theory is often applied to identify critical habitat and to aid in reserve design. In these cases, empirical measures of dispersal behaviour and patterns that increase realism along with realistic connectivity indices may be of utmost importance.

## 5. Conclusion

Hilltopping is a common behaviour for many butterfly species and other insects. In the present study, we provide the first empirical evidence of an effect of hilltopping on spatial population dynamics. We found evidence supporting the inclusion of empirically based connectivity metrics that incorporated hilltopping behaviour over those that did not. Moreover, population models including density dependence and the interaction of temperature and precipitation were favoured over those that were less complex. In simulations of spatial dynamics based on model fits with different dispersal modes, we found that models with hilltopping were closer to observed patch dynamics and had lower occupancy and regional synchrony, and higher extinction and colonization rates. These patch characteristics could affect the probability of regional persistence, depending on the balance of local extinction and colonization rates versus regional synchrony. Overall, our study suggests the importance of incorporating ecological realism, in this case non-random dispersal, when modelling the dynamics of a spatially structured population in an increasingly fragmented world.

## Data Availability

All data and code associated with this study are available on Zenodo at https://doi.org/10.5281/zenodo.6354208 [44]. Electronic supplementary material is available online [45].
